# Smear layer removal in canals shaped with reciprocating rotary systems

**DOI:** 10.4317/jced.51170

**Published:** 2013-12-01

**Authors:** Paula Amaral, Leopoldo Forner, Carmen Llena

**Affiliations:** 1 Clínica Odontològica. Universitat de València, Valencia

## Abstract

The aim was to assess the presence of smear layer after canal instrumentation with two reciprocating rotary systems and a continuous motion one. Thirty canals were shaped with Reciproc, WaveOne or Mtwo systems. Smear layer was assessed following a three value scale at coronal, middle and apical levels with a scanning electron microscopy. Reciproc scores: coronal third, 20% of the cases: 0, 60%: 1, 20%: 2; middle third, 10%: 0, 20%: 1, 70%:2; apical third: 2 in all cases. WaveOne scores: coronal third, 0 (40%), 1 (30%) and 2 (30%); middle third, 0 (20%), 1 (50%), 2 (30%); apical third, 0 (20%), 2 (80%) of cases. MTwo scores: coronal third 0 (50%), 1 (30%) 2 (20%); middle third 0 (20%), 1 (50%), 2 (30%); apical third, 0 (10%), 1 (10%), 2 (80%). No significant differences (p>0.05) were found between the three used systems.

** Key words:**Endodontics, reciprocating motion files, rotary file, SEM, smear layer.

## Introduction

Mechanical and biological goals of root canals treatment are properly cleaning and shaping them, removing all the pulp tissue, bacteria and their products, as well as giving a suitable conformation for subsequent sealing (1,2). Therefore, mechanical instrumentation of canals is essential in endodontic treatment, due to the fact that its conformation helps in canal disinfection through irrigation (3). The introduction of nickel and titanium alloys (NiTi) promoted the emergence of instruments with a continuous rotary motion inside the root canal, usually with a crown-down technique. Reciprocating rotary motion systems such as Reciproc (VDW, Munich, Germany) and WaveOne (Maillefer, Ballaigues, Switzerland) as one-file systems have recently appeared, with the aim of reducing the number of steps and files to reach a correct endodontic treatment. They are both made with Mwire alloy (NiTi), which provides more flexibility, greater resistance to cyclic fatigue and better handling of curved, narrow and deep canals (4-7), than the traditional NiTi alloy manufactured instruments, although some controversy exists in this regard (8), looking for a decrease in the breaking of instruments, failures due to torsional or flexural fatigue (9). A canal preparation procedure based on the balanced forces technique, is used for these reciprocating motion rotatory systems.

The objective of this study was to determine the ability of these new files in cleaning the canal internal surface, trough smear layer measurement after instrumentation with them and with a continuous motion rotatory system.

## Material and Methods

- Sample selection

The sample consisted of mandibular molars (N=30) with root curvatures between 20°-25° (angle between the tooth axis and the line joining the apex to the beginning of the curvature formation). Roots were detached from crowns. The study only included teeth with a distal canal and without open apex, resorption or calcifications. The working length was determined subtracting a millimetre when a size 15 K-file was visible through the apical foramen. Molars were randomly distributed into 3 groups (ni=10) as described below.

- Canal instrumentation

Samples were prepared with three rotatory systems: group 1, Reciproc; group 2, WaveOne; and group 3 Mtwo.

Reciproc is a rotatory system with reciprocating motion, only using a file for canal preparation. The system provides three files with the following features (tip diameter and taper): R25 (25/0.08), R40 (40/0.06) y R50 (50/0.05). WaveOne is also a one-file system with rotatory reciprocating motion, offering three files: “Small” (21/0.06), “Primary” (25/0.08) and “Large” (40/0.08). MTwo is a continuous rotary motion system with four basic instruments: 10/0.04, 15/0.05, 20/0.06 y 25/0.06 and other three complementary to widen the apical third: 30/0.05, 35/0.04, 40/0.04. First and third instrument sections were a double S, cutting simultaneously at two points in the canal walls, whereas the second one was a triangle with convex walls, thus touching at three points.

Reciprocating motion rotatory instruments prepared the coronal third, firstly, after that, the middle third was prepared, both with short back and forward movements, removing the file after every three movements of this type. Working length was confirmed before the apical third was prepared. All the MTwo files work at working length, following the so called “simultaneous technique”, thus the working length is achieved with the first file; in this system instruments are used in an ascending order, referred to the tip diameter.

In all groups, canals were irrigated with sodium hypochlorite 5.25% every time the file was pulled, and this in turn was cleaned with an alcohol pad. An 18% EDTA solution was used as final irrigant for 3 minutes, and after that, 3 ml of saline were applied.

- Smear layer observation.

**Figure 1:**
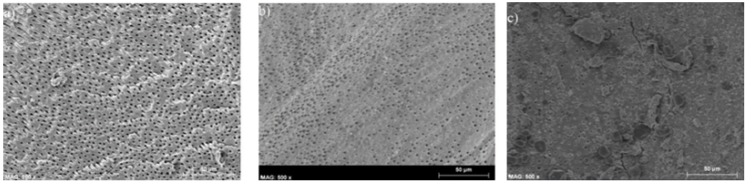
Examples of the different scores used –canals shaped with MTwo-: a) no smear layer (coronal third);b) smear layer and some open tubules (middle third); c) a big amount of smear layer in the apical third, only few tubules can be seen.

Grooves were performed with a diamond bur in the vestibular and lingual surfaces of all distal canals. With this, and with the aid of a dental chisel, each root was separated into two halves, which were placed in suitable supports. Specimens were then metallized with a gold-palladium layer and observed with an emission field scanning electron microscopy Hitachi S-4100 at 500x. Smear layer presence was assessed through Torabinejad classification (10), in a three value scale (Fig. 1). When canals presented a high smear amount, covering dentine and dentinal tubules surface, a score of 2 was given; score 1 was for canals with relatively clean surface but with moderate smear inside the tubules; finally, score 0 was given when canals and tubules were free of smear layer (11,12). Observations were performed in the central area of coronal, middle and apical thirds in each root.

- Statistical analysis.

A κ index was performed in order to establish an intra-observer error. For data analysis, the χ2 test was used with a significance level of p>0.05.

## Results

**Figure 2:**
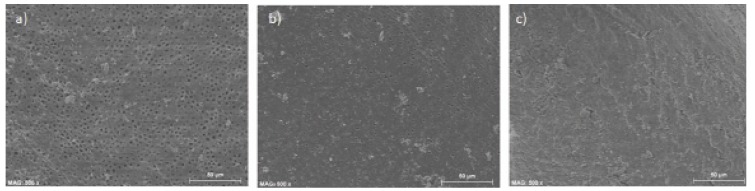
Reciproc. Examples of the most prevalent images in the different thirds (coronal -a-, middle -b- and apical .c-) -scores: 1, 1, 2, respectively.

For Reciproc system, scores for the used scale were as follows (Fig. 2). Coronal third: 60% of cases, score 1; 20% 2 and 20% with no smear layer. Middle third: 70% of specimens got score 2, 20% 1 and 10% 0. In the apical third smear was abundant in all cases.

**Figure 3:**
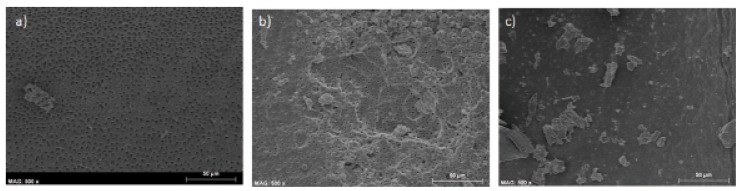
WaveOne. Examples of the most prevalent images in the different thirds (coronal -a-, middle -b- and apical .c-) -scores: 0, 1, 2, respectively.

For WaveOne system (Fig. 3), in the coronal third, 40% of cases showed score 0, 30% score 1 and 30% score 2; for the middle third, 50% of cases presented moderate smear, 20% had no smear at all and 30% presented a high amount of smear; apically 80% showed score 2 and 20% 0.

The MTwo system showed the following values of smear (Fig. 1): coronal third, 50% value 0, 30% value 1 and 20% value 2; middle third, 20% value 0, 50% value 1 and 30% value 2; apical third 80% value 2, 10% value 1 and 10% value 0.

When only alternate motion systems were considered, for the coronal third, value 0 appeared in 42.9% of cases for the Reciproc system and in the remaining 57.1% for the WaveOne; value 1 was present in 66.7% and in 33.3% respectively, whereas value 2 was in 42.9% and in 57.1% respectively; in the middle third, value 0 supposed a 33.3% of cases for Reciproc and a 66.7% for WaveOne; value 1 was for 28.6% of cases and 71.4% respectively, and value 2 was for 70% and 30% of cases respectively; finally, in the apical third a 100% of WaveOne cases had 0 value, and value 2 was observed in 44,4% of WaveOne cases and in 55.6% of Reciproc ones.

No significative differences (p>0,05) were found for the three used systems, and neither between the two one-file reciprocating motion systems. However, concerning the elimination of smear layer, better results were obtained in the coronal and middle third, compared to the apical third, where a high amount of debris was observed.

## Discussion

In the present study, the cleaning ability of dentinal walls was assessed for three rotatory systems designed for shaping root canals. Two of the systems works in reciprocating motion, WaveOne and Reciproc, whereas the third one, MTwo, presented continuous rotary motion. The smear layer removal in root canals had been analyzed for other one-file systems, as self-adjusting files (13,14), also using an F2 Protaper system file (Maillefer, Ballaigues, Switzerland) with reciprocating motion (1), and comparing the former with a complete Protaper sequence (15). It was made evident that the quality of canal cleaning depended on canal morphology and not on the type of instrument used, with no significant differences between instruments in round canals, but with better results with the complete sequence of Protaper in oval ones. The influence of the operator and the number of uses of each instrument had also been studied (9,16).

Another important aspect is possible dentinal debris extrusion through the apex. A recent study comparing reciprocating motion systems (Reciproc and WaveOne) with those with continuous motion, ProTaper and MTwo (17,18), concluded that MTwo and Reciproc showed the best results concerning smear layer removal, and obtaining the same results for debris elimination. In our study Reciproc was the system generating the highest amounts of smear.

Smear layer removal observation with scanning electronic microscopy, as used in our work, is a common technique in these kind of studies (10,19-21). Torabinejead classification, based in a three value range (0, 1 and 2) is very similar to Rome classification, and both of them have been used in several studies (13,14). Magnification used in these studies, ranged from 15x to 2500x. High amounts of smear can be observed at low magnifications, but details as waste remnants or the entire dentinal tubules must be seen at a higher magnification. However, the disadvantage of using large magnifications is the decrease of the assessment area, and therefore the performance of very limited observations, hence the decision to use 500X for this study (10).

Our results are very similar to those of other studies with MTwo and Protaper, where a higher amount of smear was also observed in the apical third, whereas coronal and middle thirds showed more clean dentine (22). Sodium hypochlorite followed by EDTA, is the treatment with the greatest impact on smear layer (19), thus it has been used as irrigation procedure in our work.

We can conclude that the three canal preparation techniques studied, Mtwo, WaveOne and Reciproc, are effective in smear layer removal, mainly in the middle and coronal thirds, without significant differences between them.
